# Human Preferences for Symmetry: Subjective Experience, Cognitive Conflict and Cortical Brain Activity

**DOI:** 10.1371/journal.pone.0038966

**Published:** 2012-06-13

**Authors:** David W. Evans, Patrick T. Orr, Steven M. Lazar, Daniel Breton, Jennifer Gerard, David H. Ledbetter, Kathleen Janosco, Jessica Dotts, Holly Batchelder

**Affiliations:** 1 Program in Neuroscience, Bucknell University, Lewisburg, Pennsylvania, United States of America; 2 Geisinger Health System, Danville, Pennsylvania, United States of America; Institute of Psychiatry at the Federal University of Rio de Janeiro, Brazil

## Abstract

This study examines the links between human perceptions, cognitive biases and neural processing of symmetrical stimuli. While preferences for symmetry have largely been examined in the context of disorders such as obsessive-compulsive disorder and autism spectrum disorders, we examine various these phenomena in non-clinical subjects and suggest that such preferences are distributed throughout the typical population as part of our cognitive and neural architecture. In Experiment 1, 82 young adults reported on the frequency of their obsessive-compulsive spectrum behaviors. Subjects also performed an emotional Stroop or variant of an Implicit Association Task (the OC-CIT) developed to assess cognitive biases for symmetry. Data not only reveal that subjects evidence a cognitive conflict when asked to match images of positive affect with asymmetrical stimuli, and disgust with symmetry, but also that their slowed reaction times when asked to do so were predicted by reports of OC behavior, particularly checking behavior. In Experiment 2, 26 participants were administered an oddball Event-Related Potential task specifically designed to assess sensitivity to symmetry as well as the OC-CIT. These data revealed that reaction times on the OC-CIT were strongly predicted by frontal electrode sites indicating faster processing of an asymmetrical stimulus (unparallel lines) relative to a symmetrical stimulus (parallel lines). The results point to an overall cognitive bias linking disgust with asymmetry and suggest that such cognitive biases are reflected in neural responses to symmetrical/asymmetrical stimuli.

## Introduction

Humans appear to have an inherent appreciation for many symmetrical aspects of the natural world, such as markings on coral reef fish and butterflies [1]. Non-humans also appear to recognize and prefer symmetry. Honey bees (*Apis mellifera*), for example, show preferences for flowers displaying radial symmetry [2]. Preferences for symmetry have been attributed to a variety of evolutionary pressures that equate symmetrical signaling systems with constructs such as beauty, attraction, and biological fitness [3]–[6]. The human appreciation for symmetry appears to go beyond signaling and the evaluation of biological fitness, extending to a more general sense of aesthetics.

Work with human infants demonstrates that children begin to show preferences for vertical symmetry by four months of age [7], a preference which is well-established by twelve months of age [7]. More recent work in humans has focused almost exclusively on the link between beauty, attraction, the preference for symmetrical faces, and the impact of this preference on sexual selection [8], [9]. Given the apparent, universal proclivity to prefer symmetry in human and non-human animals, preferences for symmetry could represent important evolutionary impulses. Symmetry preferences exist in a wide range of animals despite the fact that visual systems have evolved differently across species (e.g., the visual systems of cephalopods and insects are markedly different in structure and function from the human visual system [1]).

Although symmetry preferences are adaptive, some neurodevelopmental disorders are marked by symptoms involving restricted interests with symmetry and behaviors involving checking, ordering and arranging objects in straight lines or symmetrical patterns [10]. Persons with autism evidence sensitivity to symmetry [11] and exhibit behaviors involving lining up objects into symmetrical patterns. Obsessive compulsive disorder (OCD) is marked by troubling intrusive thoughts, images or impulses (obsessions) and repetitive, circumscribed behavior patterns [12]. The behavioral presentation of OCD is highly variable [13], but obsessions often include preoccupations with symmetry. Compulsions may include checking, washing, ordering and arranging, counting, touching or tapping, and repeating elaborate rituals or routines [12]. Because of the broad and varied behavioral phenotype of compulsive behavior and the increased characterization of autism as a “spectrum” disorder, there is a growing appreciation for regarding autistic-like and OC behavior from a dimensional perspective, rather than the more traditional dichotomous approach to neuropsychiatric disorders [13].

Not only are disorders such as OCD and autism increasingly characterized as comprising a spectrum, many of the behaviors associated with these disorders are highly prevalent in the general population [14]–[16]. Nearly 90% of the general population report some obsessions, preoccupations or compulsive urges involving symmetry. The content of these normative variants of OC behavior is strikingly similar to those observed in OCD and autism, differing only in the frequency, intensity and the degree to which intrusive thoughts may be dismissed, or behaviors resisted [15], [16]. The prevalence of preoccupations, restricted interests and repetitive behavior is sufficiently high in the general population that some have suggested that such behaviors may be well-conserved vestiges of once adaptive behaviors rooted in our phylogenetic history [17]–[22].

Given that autism spectrum (ASD) and OC behaviors are common in the general population, typical and atypical variants may reflect similar cognitive and neuropsychological themes. For example, patients with OCD/ASD exhibit deficits on executive function (EF) tasks requiring planning, attention, cognitive set-shifting, and response suppression [23], [24]. One common EF deficit in OCD/ASD involves the Stroop test [25], in which subjects are asked to read a list composed of color names. Subjects are asked to ignore the word itself and identify the color ink that the word is printed in, which is inconsistent with the word itself (for example, the word “red” may be printed in green ink). Subjects with OCD have particular difficulty on the Stroop task [26] and on set-shifting tasks, such as the Wisconsin Card Sort Task (WCST) [24], [27], which requires inhibiting previously reinforced responses. First degree relatives of subjects with OC also perform worse on the WCST relative to control subjects. Such findings highlight the endophenotyic nature of brain-behavior links in the OC spectrum [27].

Recent variants of the traditional Stroop task add an emotional valence to the stimuli. For example, using words like “murder” or “cancer” to evoke an affective response may enhance the Stroop or task-switching effect. These emotional variations of traditional neuropsychological tasks may be particularly useful in studies involving anxiety. In assessing OC spectrum behaviors in a Stroop-like task, experimenters may use words such as “germs” or “dirt” to evoke an emotional response that creates greater cognitive interference than neutral words (such as color names). Indeed, patients exhibiting OC symptoms show a greater cognitive and attentional bias for negatively valenced stimuli that are relevant to their OC symptoms [28].

Such executive control deficits are believed to be governed by certain cortical and subcortical regions that comprise the cortico-striatal-thalamo-cortical loop. This loop has been reliably implicated in the pathogenesis of OCD in both structural and functional studies [29]–[32]. Performance on an affective/high-contrast Stroop was associated with enhanced N200 Event-Related Potential (ERP) waveforms in OCD patients [33]. The N200 is associated with task switching, uncertainty, conflict monitoring and inhibition [34]. Such activity tends to be localized frontally along the midline, over scalp sites associated with the dorsolateral and anterior cingulate cortices.

Among the more consistent imaging findings in OCD is that patients exhibit atypical cortical activity on “Oddball” tasks [35], which present subjects with a standard, or frequently occurring stimulus (approximately 80% of the trials) with presentation of a rare or deviant stimulus on a random schedule on 20% of the trials. Specifically, subjects with OCD exhibit more pronounced (i.e., greater amplitude) and faster (shorter latency) P300 responses when presented with changes in a stimulus [36]. Greater amplitude and faster processing in OCD subjects is believed to reflect the over-focused attention and faster cognitive processing that is typical in OCD subjects [36]. Subjects with ASD also appear to exhibit atypical cortical responses on visual oddball tasks [37]–[41]. The sensitivity, and resistance, to change that is a core feature of the ASD and OC phenotypes is likely associated with an oddball task of symmetry perception.

In this study we attempt to link various behavioral levels of analysis associated with sensitivity to and preferences for symmetry. We examine the shared variance between self-reports of obsessive-compulsive behavior, cognitive and affective associations of symmetry on a Stroop/Implicit Association task developed to assess implicit cognitive biases linking images of faces depicting certain emotions (disgust in this case) with words associated with asymmetry. Finally, we utilize and oddball stimulus specifically designed to assess sensitivity to symmetry. We found that subjective reports of preferences for symmetry and order are linked to cognitive biases on this adaptation of the Implicit Association Task, which, in turn, is linked with cortical processing of an asymmetrical oddball stimulus.

## Materials and Methods

### Ethics Statement

The research protocol and consent procedures were approved by the Institutional Review Board of Bucknell University (IRB #1112-033, “Social Cognition and Face Perception”). All subjects involved in the study gave written informed consent. Written parental consent (and oral assent) was obtained for the sole 17-year-old participant.

### Statistical Analysis

All analyses were performed using SPSS 19 (IBM) with a significance threshold set at 0.05. Variable distributions were checked for normality, and non-normally distributed variables were analyzed using non-parametric statistical tests, as noted in the results.

### Experiment 1

Subjects were undergraduates at a liberal arts university in central Pennsylvania (N = 82; 19 male; 63 female). Subjects completed a demographic form with information on gender, race, date of birth, ethnicity, religion and psychiatric history. All participants had normal to corrected-normal vision and normal color vision. Subjects' participation served as an option for satisfying partial research credit in an introductory psychology course. Subjects ranged in age from 17 to 21 years of age (*M* = 18.75; *SD* = .73). Subjects were administered a series of computer-generated tasks and inventories.

All subjects were administered a computer version of the Obsessive Compulsive Inventory (OCI) [42]. The OCI contains 42 items comprising seven subscales (Washing, Checking, Doubting, Ordering, Obsessing, Hoarding, Mental Neutralizing). Each item was rated on a five-point Likert scale. For each item, subjects are asked to rate the frequency and the degree of distress that each item presents to the participant. The OCI has excellent internal consistency and has been shown to discriminate between subjects with and without OCD diagnoses.

Subjects were administered a computer-generated adaptation of the Implicit Association Task (OC Cognitive Interference Task, or OC-CIT). This task required subjects to sort words associated with symmetry/asymmetry and images of facial emotional expressions of happiness/disgust (Nim-Stim) [43]. Subjects were presented with a series of words that either represent symmetry (Symmetrical, Balanced, Straight, Arranged, Aligned) or asymmetry (Jumbled, Cluttered, Irregular, Scattered, Crooked). Using the “E” and “I” keys (Left and Right), subjects were instructed to sort words reflecting one of the two categories, as quickly as they could, while making as few mistakes as possible. The first set included 20 trials, followed by 40 trials. Next subjects were asked to sort faces representing Disgust or Happiness (see Figure S1) for 20, then 40 trials. In the final set, subjects were asked to indicate (again with “E” or “I” keys) whether a stimulus was *either* a word reflecting symmetry/asymmetry *or* a Disgust/Happy face. Subjects were asked to sort a “congruent” (Happy with Symmetry; Disgust with Asymmetry) block of stimuli and an “incongruent” (Happy with Asymmetry; Disgust with Symmetry) block of stimuli (Figure S1).

In Experiment 1, we aimed to determine the link between the cognitive interference on the incongruent matching trials (relative to the congruent trials) with subjective reports of OC behavior on the OCI. We did so in two separate phases of testing. Subjects in phase 1 (n = 27) received the congruent tasks first, followed by the incongruent tasks whereas subjects (n = 55) in phase 2 received the incongruent tasks first, followed by the congruent tasks. Thus, we are able to examine whether the incongruent block yields more errors and longer response latency relative to the congruent block apart from the expected perseveration of a cognitive set-shifting task. That is, we sought to determine whether a) there was an implicit association indicating preferences to match the Happy/Symmetry-Disgust/Asymmetry condition; b) whether such associations were demonstrated to be statistically more likely regardless of order of presentation, and c) whether the degree of interference is associated with the subjective experiences of OC behavior – particularly regarding reports of Ordering behavior on the OCI.

### Experiment 2

In Experiment 2 subjects were 26 undergraduates (19 female; 5 male) from the same University and same data pool (from a section of Introduction to Psychology course). Experiment 2 aimed to examine the link between the cognitive and affective associations with symmetry as measured by the OC-CIT, and cortical brain activity on an ERP oddball paradigm task designed specifically to measure sensitivity to symmetry [31].

Two oddball tasks were administered. The “Spheres task” is a traditional visual oddball task consisting of an image of a blue sphere presented on 80% of the trials (the standard stimulus), with a red sphere presented on 20% of the trials (the deviant stimulus) on a random schedule. Both spheres measured 6.2 cm in diameter. Images were presented on the screen for one second, with an inter-stimulus interval of 300 ms. The task lasted for four minutes. After a 1 minute break, subjects were then presented with the second oddball task (the order of the tasks were randomized). The parallel lines task presented subjects with a standard stimulus (80% of trials) consisting of pair of yellow parallel lines on a black background. The lines measure 11.25 cm×1.5 cm separated by 3.75 cm (See Figure S2). The deviant stimulus (20% of trials) consisted of an identical set of lines, except that the line on the right was rotated at a 9° angle (Figure S2). These stimuli were also presented for one second with an inter-stimulus interval of 300 ms. Stimuli were presented on a Dell computer monitor (16 in).

Event-related potentials were recorded using a 32 channel amplifier, Ag-AgCl electrode fabric cap arranged in the international 10–20 system, grounded at site AFz (ANT, Enschede, Netherlands). Signals were recorded at a sampling rate of 512 Hz, filtered continuously with a high pass of .3 and a low pass of 30 (as recommended by ANT). Averaging epochs were set to −0.1 seconds before, to 0.6 seconds after stimulus presentation. Impedances were maintained below 10 KΩ. ERP data were analyzed with ASA (Advanced Source Analysis) version 4.7.3. Analyses of the averaged waveforms were identified for early (125 ms–265 ms) and late (270–600). Date were obtained for both oddball tasks by recording the latencies and mean amplitudes (both positive and negative) both pos amplitudes of the highest peaks of standard and deviant conditions occurring between a 200 ms and 500 ms window. Electrode sites (F3, F7, F4, F8, Cz P7, P8) were used for analysis and represent left and right dorsolateral, midline and parietal sites. We analyzed site Pz as a protoypical P300 averaged waveform to verify that the parallel lines task was consistent with other oddball tasks.

## Results

### Experiment 1

**Figure 1:**
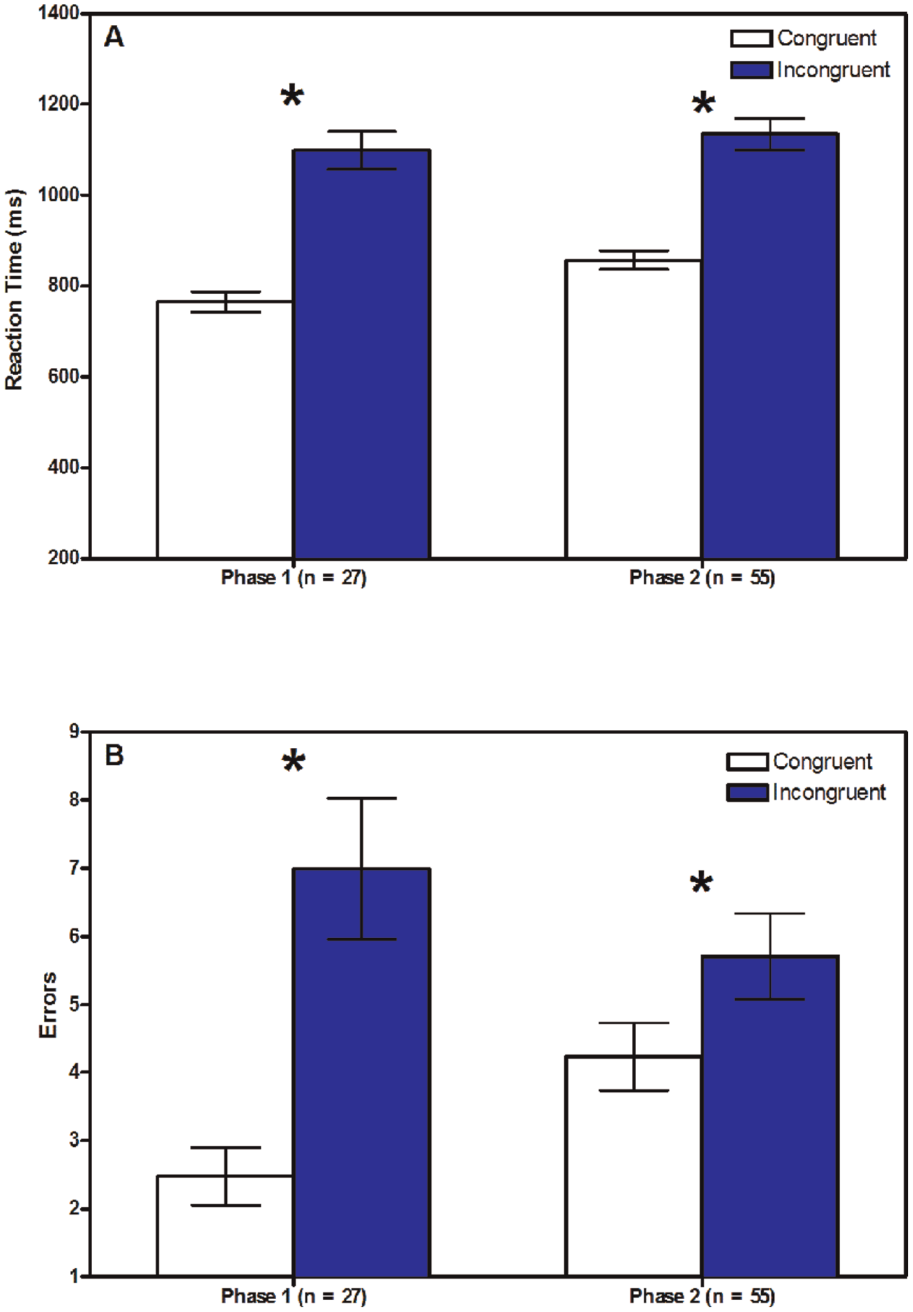
Differences in Performance on the OC-CIT. Subjects had significantly higher reaction times (A) and made significantly more errors (B) during the congruent sorting task than during the incongruent sorting task, regardless of the ordering of these tasks. Bars represent mean ± standard error. Asterisks indicate a significant (*p*<0.05) difference between the bars for the congruent and incongruent tasks.

A 2 (phase condition: congruent- or incongruent-first)×2 (congruent/incongruent Reaction Time) repeated measures analysis of variance was performed. The interaction effect was not significant (*p*>.10), but the main effect of congruity on reaction time (RT) was significant (*F*(1,80) = 244.05, *p*<.0001; Figure 1A), indicating that the mean RT for the incongruent condition (*M* = 1122.57 ms, *SD* = 242) was significantly longer than for the congruent condition (*M* = 826.39 ms, *SD* = 146), regardless of the order in which the two tasks were presented. Since reaction time was non-normally distributed, the effect of congruity was re-analyzed using the non-parametric Related Samples Wilcoxon Signed Rank test, which indicated a significant effect for congruity (*Z* = −7.861, *p*<.001). Thus, these data indicate that subjects exhibit greater facility with the congruent condition than the incongruent condition, beyond an effect of set-shifting.

A similar repeated measures ANOVA was performed for number of errors. The main effect for congruity was significant (*F*(1,80) = 33.07, *p*<.0001; Figure 1B), indicating that more errors in the incongruent (*M* = 6.1, *SD* = 4.91) than in the congruent (*M* = 3.66, *SD* = 3.36) conditions, regardless of phase. This effect was confirmed using the non-parametric Related Samples Wilcoxon Signed Rank test (*Z* = −4.907, *p*<.001). However, there was a significant interaction effect between phase and congruity (*F*(1,80) = 5.49, *p*<.05). Despite this, in Phase 2, subjects still made significantly more incongruent than congruent errors (*p*<.05) indicating greater cognitive interference in the incongruent condition.

Finally, the frequency scores from the OCI were entered into a multiple regression equation in order to determine which OCI scales account for variance in the difference between the incongruent and congruent reaction times (Incongruent RT – Congruent RT). Consistent with expectations, the Checking subscale of the OCI was the sole predictor of the Incongruent-Congruent RT difference scores, accounting for 13 percent of the variance (*F*(1,80) = 11.49, *p* = .001). Similar multiple regressions were performed for the difference between incongruent and congruent Errors. The Washing OCI subscale predicted 6 percent of the variance (*F*(1,80) = 5.05, *p* = .027) and no other OCI variables predicted additional significant variance.

### Experiment 2

**Figure 2:**
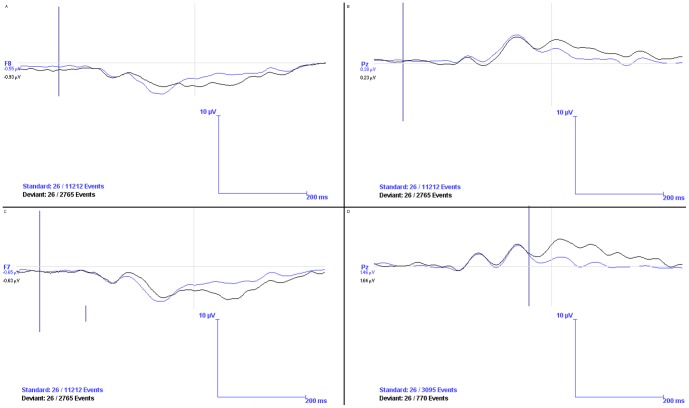
Grand Averages of Selected ERP Waves. During the parallel lines oddball task, at electrode sites F7 (A) and F8 (C) there was an early period of relatively more positivity followed by a later period of relatively more negativity in response to the deviant stimulus, as compared to the standard stimulus. There was increased positivity in response to the deviant stimulus, as compared to the standard stimulus, at electrode site Pz during both the parallel lines (B) and spheres (D) oddball task.

As Figure 2 indicates, at Pz the Lines task resulted in an expected P300 effect, with the deviant (unparallel) condition evoking a significantly more positive peak and mean amplitude than the standard (parallel) condition. Next, we determined the rates of processing (latencies) of the parallel and unparallel conditions of the ERP task at all candidate electrode sites for both early and late components. The mean and peak amplitudes were significantly different between standard and deviant conditions at all electrode sites. Latencies were similar between standard and deviant conditions, except for P8, where the deviant (unparallel) condition was processed faster than the standard (parallel) condition (*t*(25) = 2.35, *p*<.05) at the early, negative component (see Figure 2).

**Figure 3:**
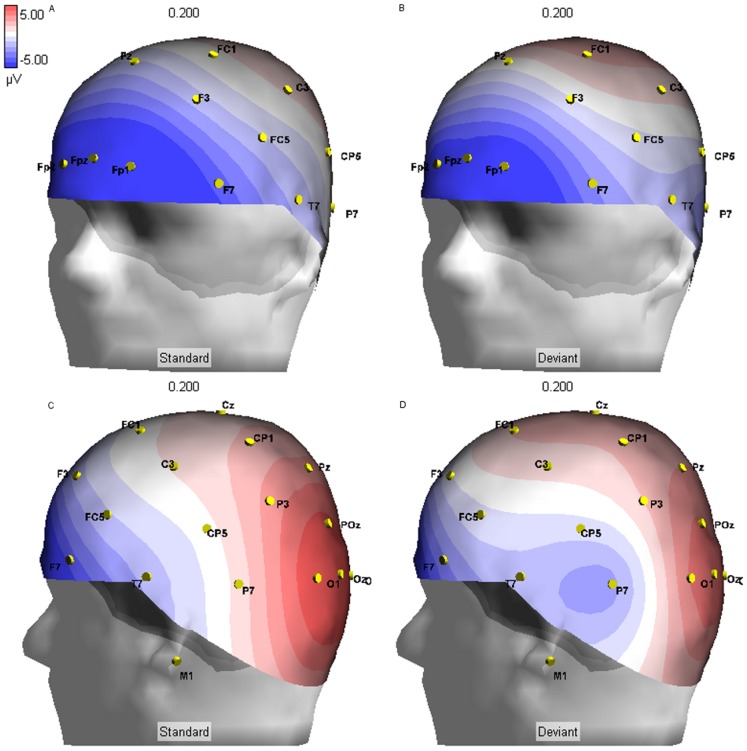
ERP in Response to the Parallel Lines Oddball Task, 200 ms post-stimulus. 200 ms after stimulus presentation, amplitude at site F7 is relatively more positive after the deviant stimulus (B), as compared to the standard stimulus (A). Negativity is observed at electrode site P7 after the deviant stimulus (D), but not after the standard stimulus.

**Figure 4:**
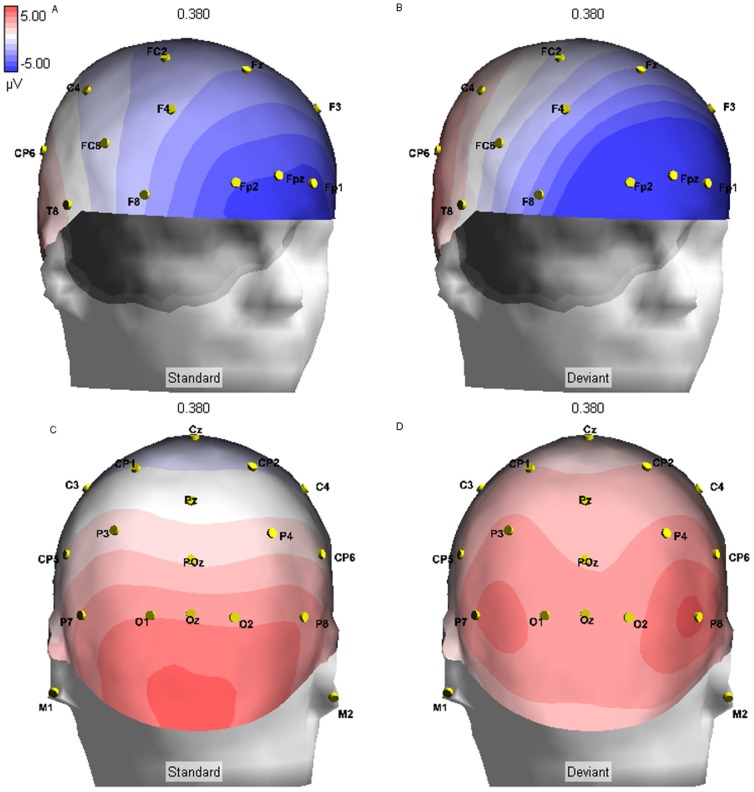
ERP in Response to the Parallel Lines Oddball Task, 380 ms post-stimulus. 380 ms after stimulus presentation, amplitude at site F8 is relatively more negative after the deviant stimulus (B), as compared to the standard stimulus (A). Amplitude at site Pz is relatively more positive after the deviant stimulus (D), compared to the standard stimulus (C).

Three-dimensional renderings were generated for standard and deviant conditions. We note greater relative positivity under the deviant condition relative to standard at frontal sites for the early (120–265) ERP component (Figure 3). During this same time period, we observed focal, bilateral negative amplitudes centered at sites P7 and P8 under the deviant condition, which was not observed for the standard condition. There were significant differences in maximal deflection from zero at sites P7 (*t*(25) = −6.497, *p*<.001) and P8 (*t*(25) = −4.164, *p*<.001) in the deviant (P7: *M* = −2.932, *SD* = 3.27; P8: *M* = −2.209, *SD* = 3.00) relative to the standard (P7: *M* = 0.019, *SD* = 2.05; P8: *M* = −0.374, *SD* = 2.39) condition during this time window. Between 270 and 600 ms after stimulus presentation, the dominant features under both stimulus conditions were frontal negativity and parietal positivity (Figure 4). However, under the deviant condition, frontal negativity during this time period was more widespread for the deviant condition. Although amplitudes were similar for both conditions at sites FPz, FP1, and FP2, there was relatively more negativity at sites F7 (*t*(25) = 3.974, *p* = .001) and F8 (*t*(25) = 3.181, *p* = .004) under the deviant condition (F7: *M* = −2.571, *SD* = 2.03; F8: *M* = −2.604, *SD* = 1.63), as compared to the standard condition (F7: *M* = −3.206, *SD* = 1.75; F8: *M* = −3.262, *SD* = 1.40). Similarly, under the deviant condition, parietal positivity was more widespread. Compared to the standard condition (*M* = 0.768, *SD* = 1.01), mean amplitude was significantly higher at site Pz (*t*(25) = 5.914, *p*<.001) in the deviant condition (*M* = 1.738, *SD* = 1.31).

#### Predictors of the OC-CIT from ERP Components

Multiple regression analyses examined predictors of the incongruent-congruent RT difference on the OC-CIT. We predicted that increasing cognitive/affective conflict during the incongruent condition (both relative to, and independent of, the congruent condition) of the OC-CIT will be reflected in the cortical responses during presentation of an asymmetrical (unparallel lines) stimulus, relative to a symmetrical (parallel lines) stimulus. We calculated difference scores for each of the relevant electrode site that represented differences between standard and deviant conditions for both early and late potentials. Difference scores represented the differences in mean amplitudes for all electrodes except for the early P7 and P8 negativities, in which case we used the minimum amplitudes representing the nadir.

Weighted RT difference between the incongruent and congruent condition on the OC-CIT served as the criterion variable. Amplitudes differences for the electrode sites served as predictors. The difference in mean amplitude for early positivity at F7 (F7+) accounted for 24% of the variance in weighted RT difference on the OC-CIT. Further, the difference in mean amplitude at the later negativity for F8 (F8−) predicted an additional 19% of the variance, for a total of 43% of the variance (*F*(1,24) = 5.58, *p* = .002. In order to determine whether the lines task predicted the OC-CIT RT differences, above and beyond a traditional oddball task, a forward linear regression was performed, first entering the standard-deviant amplitude differences on the spheres task at Pz. Again, the entire model accounted for 43% of the variance (*p* = .005), beyond that predicted by the spheres task, which did not reach statistical significance (*p*>.05). Review of the weighted OC-CIT RT X mean amplitudes of F7+ scatterplots revealed opposite directionality for males and females. These opposing effects may have attenuated the true relationships between OC-CIT RT and F7+. Therefore, we conducted separate regressions for males and females. Despite the fact that only five males were included in this analysis, the regression was statistically significant, with F7+ accounting for significant and unique variance beyond the mean amplitude difference on the spheres task at Pz (*t*(1,4) = −29.16, *p* = .02; *β* = −.96). For female subjects F7+ and F8− accounted for 39% and 24% (respectively) of the variance (for a total 63%) in weighted OC-CIT RT (*F*(2, 18) = 15.91, *p*<.001). The directionality of β was positive.

None of the difference scores for latencies predicted variance in weighted RT on the OC-CIT. However, for OC-CIT errors (difference between errors on the incongruent and congruent conditions) the early P8− mean amplitude in response to the deviant stimulus predicted 16% of the variance (*F*(1,24) = 4.65, *p* = .04).

The directionality of the multiple regression findings indicates that the greater the difference in processing speed of the parallel and unparallel conditions (that is, the greater neural differentiation or sensory/perceptual conflict presented by the unparallel condition) the greater the difference in RT between the congruent and incongruent conditions on the OC-CIT. Similarly, the simple regression indicates that faster neural processing on the unparallel condition on the ERP task was associated with greater cognitive/affective conflict when asked to associate words reflecting asymmetry with positive affect (happy faces), and likewise, symmetry words with negative affect (disgust faces).

## Discussion

This study examined the links among the subjective experiences of certain aspects of obsessive-compulsive behavior (particularly preferences for order and symmetry), performance on tasks assessing cognitive and affective associations with symmetry, and the neural processing of (a)symmetry. In doing so, we aim to elucidate various levels of analysis involved in evaluating sensitivity to symmetry and asymmetry. Human and nonhuman preferences for symmetry are well-documented in the extant literature [1]–[6]. Ontogenetic and phylogenetic advantages for sensitivity to symmetry are balanced by an impressive literature that highlights preoccupations and concomitant behaviors that are closely associated with certain neuropsychiatric disorders, including obsessive-compulsive and autism spectrum disorders. The aim of this study was to clarify the links between subjective, behavioral, and neural processes related to a construct believed to be relevant to both human adaptation and pathology – symmetry. The present data indicate that subjective reports of obsessive-compulsive behavior were related to performance on an objective task assessing cognitive and affective associations with symmetry.

These data indicate a clear preference for linking positive (happy) facial expressions with words connoting symmetry, and negative (disgust) faces with those connoting asymmetry. Regardless of the order of presentation, subjects took longer and made more mistakes when instructed to sort incongruent information (Happy with Asymmetry; Disgust with Symmetry) than congruent information (Happy with Symmetry; Disgust with Asymmetry). This is consistent with previous work showing that patients with OCD are sensitive to tasks designed to target their OC symptoms [28] but extends these findings to non-clinical manifestations of OC behavior. That is, the OC-CIT is an EF task sensitive to OC behavior in humans who engage in such behaviors at a sub-clinical level.

Experiment 1 demonstrated that subjects appear to have cognitive bias and affective associations linking positive affect with symmetry and negative affect with asymmetry. The two phases of data collection were intended to determine whether this bias held regardless of the order of presentation. Results indicated that indeed even when the incongruent face-word pairs were presented to subjects first they made more errors and had a significantly longer response time when sorting the incongruent stimuli relative to the congruent stimuli. We focused on disgust given recent findings pointing to disgust as a core feature of OC spectrum behavior [44] reflecting underlying neural processing [45]. Tolin et al. [44] report links between several dimensions of OC behavior (washing, ordering, checking) and disgust sensitivity. While it is reasonable to imagine linking disgust with certain aspects prevalent in OCD such as germ and hygiene-related stimuli, we sought to determine whether disgust extended to other aspects of OC behavior, particularly the preference for symmetry.

The results of Experiment 1 confirmed not only that there is a bias toward linking disgust with asymmetry, but also that such links are related to the subjective experience of preferences for order (as measured by the OCI). Such findings are important as they indicate a relative integration among subjective experiences and implicit biases. Given that we tested subjects in two phases, alternating presentation of the stimuli, the data indicate that this finding is not simply attributable to set-shifting deficits, which have been noted extensively in the OCD literature. Rather they punctuate the implicit perceptual, cognitive and affective biases associating symmetry with positive affect and asymmetry with negative affect.

In Experiment 2, we demonstrated that the perceptual, cognitive and affective bias for symmetry may be rooted in neural functions. Subjects were exposed to two sets of stimuli during recording of cortical brain activity. One set of stimuli was designed to assess sensitivity to symmetry by alternating images of parallel and unparallel lines in an oddball task. The other set of stimuli comprised a standard oddball task and served as a control task. Results indicated a strong link between the implicit bias linking symmetry with positive affect and asymmetry with negative affect and the sensitivity to changes from parallel to unparallel stimuli. We have demonstrated previously that the parallel lines task is an effective method for assessing OC tendencies in children [31].

Here the data suggest that the neural responses were related to implicit biases for symmetry. The directionality of the finding indicates that as the cognitive bias for linking asymmetry and disgust increases, subjects process asymmetrical stimuli faster as measured by cortical brain activity. Moreover, this finding remained robust even when accounting for the variance contributed by a standard, control P300 task, again highlighting the salience of symmetry in the phenomenology and neural function of OC spectrum behavior.

While this study did not employ imaging techniques that are designed for accurate source localization of brain activity (such as fMRI), the pattern of findings suggests that the cortical response indicated early positive frontal and dorsolateral activity and, for female participants, the findings were bilateral. The findings for males only, should be interpreted with caution given the small sample size, although the effect was large. These findings are consistent with much of the functional neuroimaging work that implicates frontal regions in the pathogenesis of OCD, and extends these findings to OC behavior in typical subjects. OCD appears to involve complex neural circuitry, including the cortical-striatal-thalamo-cortical loops. In addition to the relatively poor source localization of EEG, EEG is also limited to cortical analyses, and thus cannot assess functioning of important subcortical structures implicated in OCD, such as the striated portions of the basal ganglia. However, given that our OC-CIT task required a motor response, it is plausible to consider the role of the striatum, and the entire cortical-striatal-thalamo-cortical circuitry in the coordination of the cognitive, affective and motor systems required to engage in the task. Such analyses require measures that accurately assess localization of function as well as subcortical involvement, which may in part define future directions for this research.

Altogether, the present multi-level analysis establishes links between multiple systems including self-perception and neural activity in preferences for symmetry. Such preferences for, and sensitivities to, symmetry provide an interesting window into patterns of behavior that define basic, well-conserved features of human and nonhuman perception, as well as complex neuropsychiatric conditions. The data presented here suggest an interesting conundrum that merits further exploration: disorders such as OCD which involves complex rituals, habits and preoccupations with symmetry, cleanliness and germ aversion, may represent extreme variants of behavior that was once adaptive and to some degree remains adaptive. OC spectrum behavior may represent vestiges of animal behavior that have emerged in sharp relief as pathological conditions, but that nonetheless involve common brain-behavior links with adaptive variants of these behaviors.

## Supporting Information

Figure S1 **OC-CIT Stimuli Presentation.** In the congruent phase (A), subjects were asked to sort words relating to symmetry and order with happy faces and words associated with asymmetry and disorder with faces expressing disgust. In the incongruent phase (B), subjects were asked to sort asymmetry/disordered words with happy faces and symmetry/ordered words with faces expressing disgust. Facial images are taken from the NimStim Face Stimulus Set [43] and used with permission.
(TIF)

Figure S2 **ERP Oddball Task Stimuli.** In a novel oddball task (A), a set of parallel lines was presented to the subjects on 80% of the trials (the “standard” stimulus), and a set of unparallel lines (the “deviant” stimulus; one line rotated 9°) was presented on 20% of the trials. Subjects subsequently experienced a standard oddball task (B), during which they were presented with a blue sphere on 80% of the trials, and a red sphere on 20% of the trials.
(TIF)
