# Supplementary figures and images for: Human Preferences for Symmetry: Subjective Experience, Cognitive Conflict and Cortical Brain Activity
Source: PLoS One. 2012 Jun 13;7(6):e38966. doi: 10.1371/journal.pone.0038966 (PMC3374766; doi:10.1371/journal.pone.0038966)

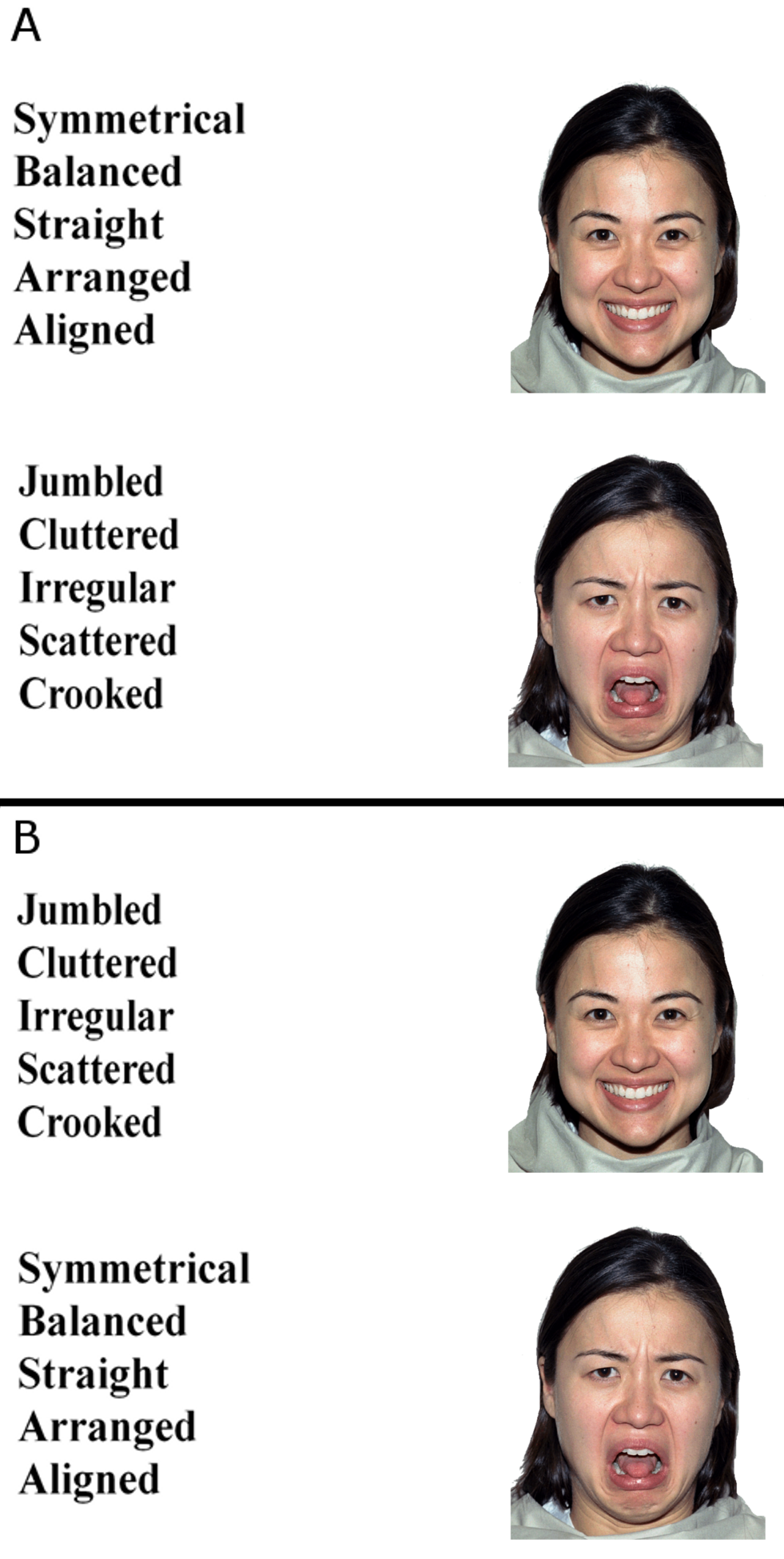

Supplement: Figure S1 — OC-CIT Stimuli Presentation. In the congruent phase (A), subjects were asked to sort words relating to symmetry and order with happy faces and words associated with asymmetry and disorder with faces expressing disgust. In the incongruent phase (B), subjects were asked to sort asymmetry/disordered words with happy faces and symmetry/ordered words with faces expressing disgust. Facial images are taken from the NimStim Face Stimulus Set [43] and used with permission. (TIF) [file pone.0038966.s001.tif]

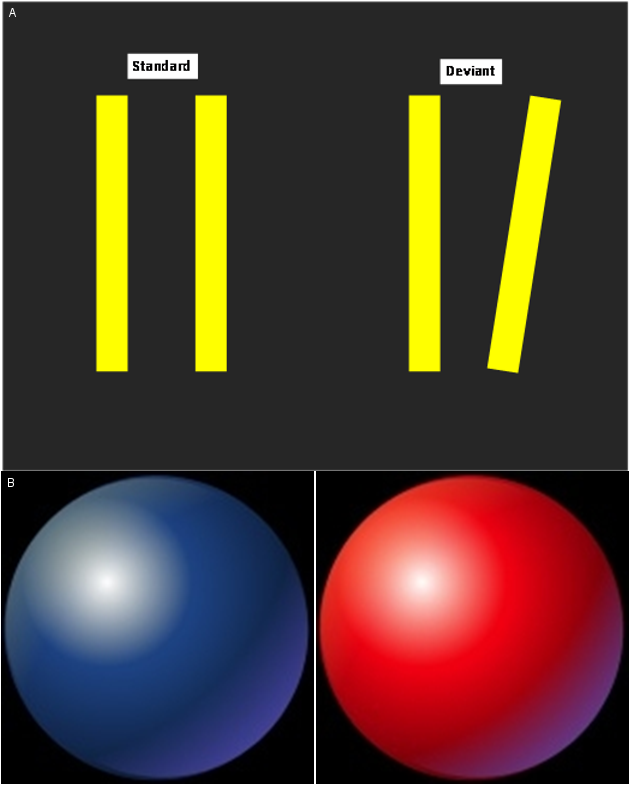

Supplement: Figure S2 — ERP Oddball Task Stimuli. In a novel oddball task (A), a set of parallel lines was presented to the subjects on 80% of the trials (the “standard” stimulus), and a set of unparallel lines (the “deviant” stimulus; one line rotated 9°) was presented on 20% of the trials. Subjects subsequently experienced a standard oddball task (B), during which they were presented with a blue sphere on 80% of the trials, and a red sphere on 20% of the trials. (TIF) [file pone.0038966.s002.tif]
